# Effect of PIVKA‐II and AFP secretion status on early recurrence of hepatocellular carcinoma after open and laparoscopic surgery

**DOI:** 10.1002/cam4.6422

**Published:** 2023-08-18

**Authors:** Yunshi Cai, Kunlin Xie, Mohammad Natheir Adeeb Alhmoud, Tian Lan, Haifeng Wan, Die Hu, Ling Lan, Chang Liu, Hong Wu

**Affiliations:** ^1^ Liver Transplant Center, Transplant Center, State Key Laboratory of Biotherapy and Cancer Center, West China Hospital Sichuan University and Collaborative Innovation Center of Biotherapy Chengdu China; ^2^ Division of Liver Surgery, Department of General Surgery West China Hospital, Sichuan University Chengdu China; ^3^ Department of Minimal Invasive Surgery Shangjin Nanfu Hospital Chengdu China

**Keywords:** AFP, hepatocellular carcinoma, laparoscopic hepatectomy, microvascular invasion, PIVKA‐II

## Abstract

**Background:**

Prothrombin induced by vitamin K absence‐II (PIVKA‐II) and Alpha‐fetoprotein (AFP) have been widely used as diagnostic markers in hepatocellular carcinoma (HCC), but the prognostic values of the two serum markers and their clinical usefulness in patient selection for different surgical approaches remain largely unclear.

**Methods:**

HCC patients received surgical treatment between 2015 and 2019 were included. Patients were divided into four statuses according to the serum PIVKA‐II and AFP secretion status: PIVKA‐II (−) AFP (−) (status 1); PIVKA‐II (+) AFP (−) (status 2); PIVKA‐II (−) AFP (+) (status 3); PIVKA‐II (+) AFP (+) (status 4). Kaplan–Meier analyses were conducted to compare the survivals of the four groups and the HCC patients received different surgical interventions; time‐dependent AUC curves were introduced to evaluate the prognostic value of the PIV‐AFP status; Cox regression model was used to identify prognostic indexes for overall survival (OS) and recurrence‐free survival (RFS).

**Results:**

A total of 518 patients were included. Patients with PIVKA‐II (+) and APF (+) presented significantly decreased OS and RFS comparing to the other statuses. The areas under ROC curves of PIV‐AFP status in predicting OS and RFS were superior to the PIVKA‐II or the AFP alone. The HCC patients in early stages with PIVKA‐II (+) and APF (+) had worse RFS when received laparoscopic hepatectomy than those who received open hepatectomy, whereas there was no difference in other secretion statuses. The PIVKA‐II (+) and AFP (+) secretion status was an independent risk factor for OS, RFS.

**Conclusions:**

The PIV‐AFP secretion status is of favorable clinical utility in predicting the OS and RFS of the HCC patients; extra caution is needed when applicated the laparoscopic approach in the HCC patients with PIVKA‐II (+) and AFP (+).

## 1 INTRODUCTION

Liver cancer remains the sixth most common cancer and the fourth leading cause of tumor‐related mortality worldwide, while the overall tumor burden from liver cancer is most pronounced in the developing countries, especially in Eastern Asia and Northern Africa.^1^ Hepatocellular carcinoma (HCC) is the dominant type of primary liver cancer, representing 75%–85% of all cases.^1^, ^2^ The prognosis of patients with HCC remains suboptimal due to frequent recurrence and rapid progression.^3^ To improve the treatment efficacies and carry out early interventions, prognostic indicators for the recurrence of HCC are needed.

Alpha‐fetoprotein (AFP) is one of the first serum markers discovered and widely used in diagnosing, surveillance and prognosis prediction in HCC.^4^ As a matter of fact, the elevated serum AFP levels are only detected in 40%–60% of all the HCC patients and 10%–20% of the early malignancies^5^; due to its inferior specificity (72%–99%) and suboptimal sensitivity (20%–65%), both the European (EASL) and the U.S (AASLD) guidelines had once removed the AFP for the surveillance of HCC.^6^, ^7^, ^8^, ^9^

Prothrombin induced by vitamin K absence‐II (PIVKA‐II), also known as des‐gamma

Carboxyprothrombin (DCP), first introduced in 1984 by Liebman et al,^10^ is used for both the diagnosis and surveillance for the HCC patients of various clinical stages mainly in Asian countries^11^, ^12^, ^13^, ^14^; despite its utility in the detection of HCC, the clinical use of PIVKA‐II in the prognosis prediction remains largely undiscussed. Microvascular invasion (MVI), defined as the presence of tumor cells in large capsule vessels or in a vascular space lined by endothelial cells,^15^ is a major risk factor for overall and recurrence‐free survival in HCC.^16^ In addition, it has been proposed by several studies that elevated serum PIVKA‐II levels and its high expression in HCC tissue are independent predictors for the histological MVI.^14^, ^17^, ^18^, ^19^

The role of the multidisciplinary modality in the management of the HCC is emerging in recent years, whereas surgical resection remains the most critical treatment. The laparoscopic hepatectomy (LH) was initially recommended for the solitary HCC with a diameter less than 5 cm or the left lateral lobectomy.^20^ On account of the improvement in surgical equipment and technology, the safety and feasibility of the laparoscopic hepatectomy increase rapidly; moreover, with the fewer postoperative complications, shorter hospital stays and comparable short‐ and long‐term outcomes comparing with a conventional open approach (open hepatectomy, OH), the interest in applying the laparoscopic hepatectomy in HCC is on the rise.^21^, ^22^ Lesion involving the major vascular structure of the liver remains the contraindication of the laparoscopic liver resection for HCC,^23^ in addition, the presence of MVI had been suggested to be an independent predictor for postoperative recurrence after laparoscopic hepatectomy.^24^

Therefore, in the current study, we aimed to (1) propose a novel classification based on the preoperative serum PIVKA‐II and AFP secretion statuses and figure out the differences in postoperative outcomes between the classifications; and (2) determine the correlation between the PIV‐AFP statuses and the different surgical approaches (OH *v. s* LH); moreover, we hoped to (3) evaluated the performance of the PIVKA‐II and the PIV‐AFP status in predicting the MVI.

## 2 METHODS

### 2.1 Study population

**FIGURE 1:**
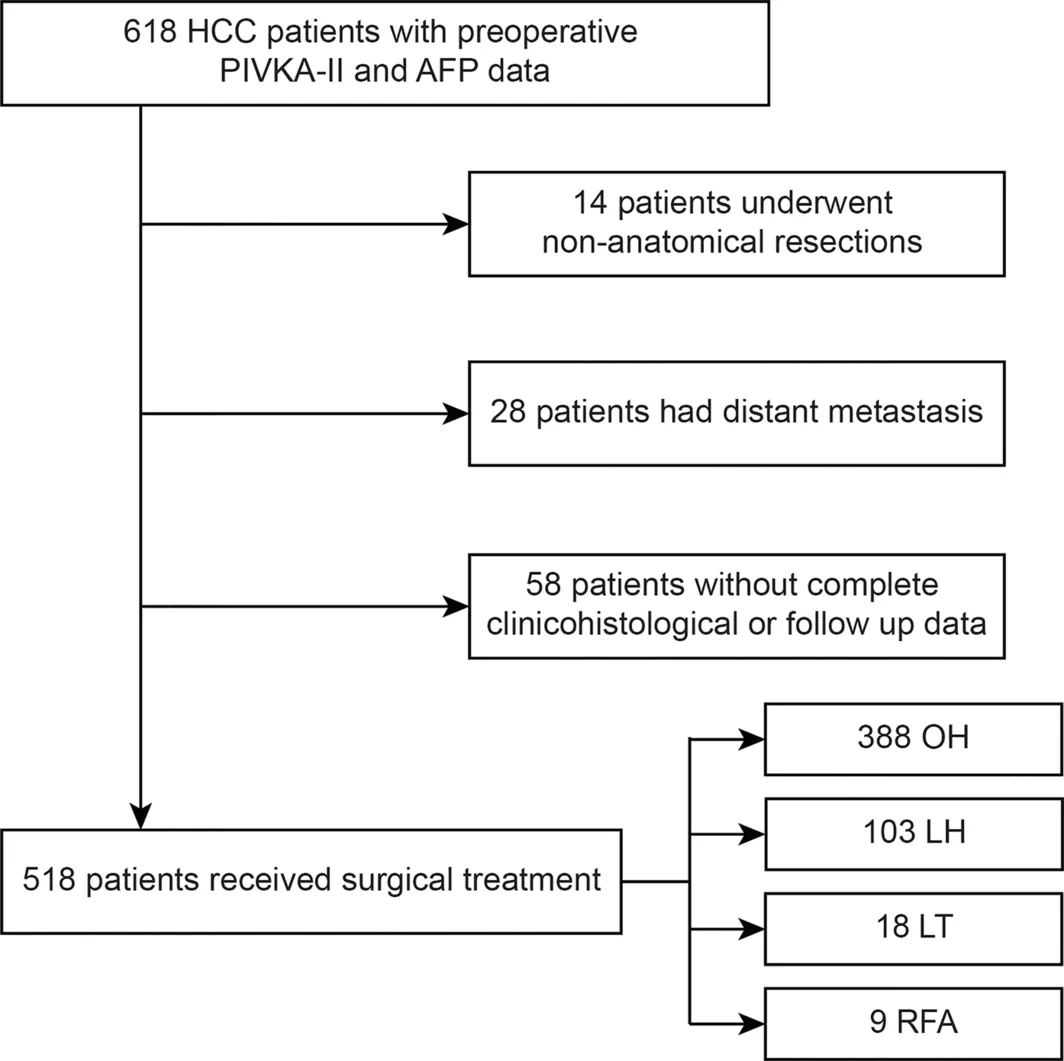
Flowchart for patient inclusion in this study.

A total of 618 patients underwent liver hepatectomy, radiofrequency ablation (RFA) or liver transplantation (LT) at the Liver Transplantation Center in the West China Hospital of Sichuan University from November 2015 to November 2019 were sequentially included. The inclusion criteria were as follows: (1) histopathological diagnosed HCC; (2) underwent surgical treatment with curative intent for the first time; (3) anatomical liver resection; exclusion criteria: (1) patients with recurrent HCC; (2) patients received preoperative antitumor treatment (transarterial chemoembolization (TACE), radiotherapy, targeted therapy, and immunotherapy); (3) distant metastasis; (4) positive surgical margin; (5) ruptured HCC; (6) impaired hepatic reserve function for liver resection; (7) incomplete clinical, histological information or follow‐up data; (8) use of anticoagulant within 2 weeks before examination of PIVKA‐II. Finally, 518 patients were included in the present study (Figure 1). All patients or their relatives provided written informed consents. This study was approved by the ethics committee of the West China Hospital of Sichuan University, in agreement with the guidelines of the 1975 Declaration of Helsinki, as revised in 2000.

### 2.2 Laboratory assays

The serum PIVKA‐II levels were determined by Chemiluminescent Enzyme Immunoassay (CLEIA) (Fujirebio Diagnostics, Tokyo, Japan, the limit of quantification: 6 mAU/mL, normal value≤40mAU/mL); the serum AFP levels were determined by Immunoassay in Electrochemistry Luminescence (ECLIA) (Roche Diagnostic GmbH, Mannheim, Germany, normal value≤7 ng/mL). The PIV‐AFP status was as follows: PIV‐AFP status 1, AFP≤7 ng/mL, PIVKA‐II≤40 mAU/mL; PIV‐AFP status 2, AFP≤7 ng/mL, PIVKA‐II > 40 mAU/mL; PIV‐AFP status 3, AFP >7 ng/mL, PIVKA‐II≤40 mAU/mL; PIV‐AFP status 4, AFP >7 ng/mL, PIVKA‐II > 40 mAU/mL.

### 2.3 Surgical procedures and techniques

Operations were sorted as follows: lobectomy (anatomical resection of the right or left hemi‐liver lobe); segmentectomy (anatomical resection of a segment); sub‐segmentectomy (anatomical resection of a subsegment, S1‐S8); liver transplantation (orthotopic cadaver liver transplantation through the piggyback maneuver or living‐donor liver transplantation); RFA (percutaneous or laparoscopic). The procedure of the anatomical liver resection was as follows: the Glissonean approach with the guidance of ultrasonography was adopted in laparoscopic or open hepatectomy, after the corresponding Glissonean pedicle was identified and temporarily occluded, the ischemic line was marked with electrocautery. Parenchyma dissection was performed using the combination of cavitron ultrasonic surgical aspirator (CUSA, Valleylab, Inc, USA) and harmonic scalpel (Ethicon Endo‐Surgery, USA) along the ischemic line and landmark vessels. Small vessels encountered were directly cut by harmonic scalpel, while for vessels≥5 mm, Hem‐o‐lock clips or titanium clips were used. The Glissonean pedicles and the main trunk of hepatic veins were secured by the linear stapler.

### 2.4 Data collection and follow‐up

All the clinical and histological data were attained from the electronic medical records (EMR) of the West China Hospital of Sichuan University. Preoperative information was obtained as follows: the total bilirubin and albumin levels; the alanine aminotransferase (ALT) and aspartate aminotransferase (AST) levels, the Hepatitis B Virus surface antigen (HBsAg) quantification, the Hepatitis B Viral loads, the PIVKA‐II and AFP levels. The AFP positivity was defined as the serum AFP >7 ng/mL; the PIVKA‐II positivity was defined as the serum PIVKA‐II > 40 mAU/mL; chronic Hepatitis B virus infection was defined as HBsAg positivity for >6 months. Clinical and histological indexes including ages and genders of the patients, numbers of tumor nodules, tumor sizes, differentiated degrees, lymph node condition, microvascular invasion (MVI), and distant metastasis were also obtained. The MVI was defined as the presence of tumor cells in vessels or in vascular space lined by the epithelial cells microscopically; the positivity of para‐aortic lymph node was regarded as remote metastasis. All included patients were staged according to the Barcelona Clinic Liver Cancer (BCLC) staging classification.^25^ Postoperation follow‐up was applied every month within 1 year, then every 3 months in the next 2 years. Thereafter, follow‐up was conducted every half year. The serum PIVKA‐II, AFP levels and enhanced ultrasound or computed tomography (CT) scan were conducted to surveil the tumor recurrence. For patients having difficulties return to the hospital for follow‐ups due to various reasons, we suggested them to exam in their local health care facilities and reviewed the findings by telephones or via the Internet. As for postoperative treatment, all HBsAg‐positive patients were given oral antiviral therapy (entecavir or tenofovir). All MVI‐positive patients were given prophylactic targeted therapy for 6 months (initially with oral lenvatinib, and if intolerable, replaced with sorafenib or regorafenib). No prophylactic TACE treatment was conducted postoperatively. At the end of follow‐up in May 2021, 58 participants dropped out due to incomplete medical records and follow‐up data.

### 2.5 Statistical analysis

EmpowerStats (http://www.empowerstats.com) and R (https://www.r‐project.org, v3.6.3) software were applicated for statistical analysis. Data were demonstrated as mean ± standard deviation (SD) or proportion. The Student's *t*‐test and Pearson's chi‐squared test were applied to compare categorical and continuous variables between groups, respectively. To assess the data with the anomalous distribution, the nonparametric Mann–Whitney U test was applied. The discriminative ability of the indexes was evaluated using the “pROC” and “survivalROC” packages in R software to calculate the area under the receiver operating characteristic curves (AUROC) or time‐dependent AUROC. The Kaplan–Meier curves were depicted based on the PIVKA‐II, AFP, and PIV‐AFP status cutoff values, and their differences between groups were evaluated by comparing the cumulative survival of the recruited HCC patients using the log‐rank test. Propensity score‐matched (PSM) analysis was performed using a logistic regression model, and it was conducted based on the nearest neighbor matching method without replacement. The following variables were enrolled in PSM: age, sex, tumor differentiation, tumor number, tumor size, and MVI.

## 3 RESULTS

### 3.1 Baseline characteristics of the patients

Five hundred and eighteen patients [436 (84.2%) males, mean (SD) age, 52.3 (19.5) years] were finally included in the cohort. The hepatitis B virus infections were observed in 441 (85.1%) of the included patients. 133 (25.7%) of the patients were with normal preoperative serum AFP levels (≤7 ng/mL), 180 (34.7%) were with high levels of AFP (>400 ng/mL). The normal levels of preoperative serum PIVKA‐II (≤40 mAU/mL) were detected in 109 (21%) of the patients; 344 (66.4%) were with high levels of PIVKA‐II (> 100 mAU/mL). The average tumor sizes in diameters were more than 5 cm (5.5 ± 3.4 cm). More than half (290, 56%) of the patients were with poor tumor differentiation. The MVI was detected in 165 (31.9%) of the enrolled patients, among which 72 (13.9%) patients were MVI more than five. Only 6 (1.2%) of the patients were with histologically verified lymph nodes metastases. A relatively small percentage (92, 17.8%) of the patients were multiple tumor nodules. The adjacent organ invasions were observed in 27 (5.2%) patients; nearly half of the patients (242, 46.7%) were with liver capsule invasions. 263 (50.8%) of the patients were stratified into the BCLC stage 0‐A, 180 (34.7%) patients were in the intermediate stage (BCLC stage B), 75 (14.5%) were with relatively advanced HCC (BCLC stage C). 388 (74.9%) of the patients received the conventional open hepatectomy; laparoscopic approaches were applied in 103 (19.9%) patients, lymph node dissections were conducted when lymph node metastases were suspected before or during the surgery; moreover, 18 (3.5%) of the patients underwent liver transplantations and 9 (1.7%) received RFAs. For postoperative treatment, 441 patients with positive HBsAg received antiviral therapy, only 142 patients out of 165 MVI positive patients received prophylactic targeted therapy, 23 patients dropped out due to drug intolerance. Patients' baseline characteristics were summarized in Table 1.

**TABLE 1:** Baseline characteristics of patients.

Characteristics	PIV‐AFP status		*p* value
	1, 2, 3 (*n* = 198)	4 (*n* = 320)	
Age, mean ± SD	53.9 ± 10.1	51.2 ± 10.7	0.003
Gender, *n* (%)			
Male	171 (86.4%)	265 (82.8%)	0.282
Female	27 (13.6%)	55 (17.2%)	
HBsAg (positive), *n* (%)	175 (88.4%)	266 (83.1%)	0.102
Tumor size (cm), mean ± SD	4.4 ± 3.0	6.2 ± 3.5	0.015
Poor tumor differentiation, *n* (%)	59 (29.8%)	169 (52.8%)	<0.001
Multiple tumors, *n* (%)	25 (12.6%)	67 (20.9%)	0.016
Lymph node metastasis, *n* (%)	0 (0.0%)	6 (1.9%)	0.087
Liver capsule invasion, *n* (%)	73 (36.9%)	169 (52.8%)	<0.001
Microvascular invasion, *n* (%)	35 (17.7%)	130 (40.6%)	<0.001
BCLC stage, *n* (%)			
0‐A	132 (66.7%)	131 (40.9%)	<0.001
B‐C	66 (33.3%)	189 (59.1%)	
Surgeries			
Laparoscopic hepatectomy	44 (22.2%)	59 (18.4%)	0.279
Open hepatectomy	141 (71.2%)	247 (77.2%)	
Liver transplant	7 (3.5%)	11 (3.4%)	
Radio frequency ablation	5 (2.5%)	3 (0.9%)	
Postoperative treatment, *n* (%)			
Antiviral therapy	175 (88.4%)	266 (83.1%)	0.102
Prophylactic targeted therapy	25 (12.6%)	117 (36.5%)	<0.001
Overall survival, month, mean ± SD	39.1 ± 20.0	28.8 ± 17.4	<0.001
Recurrence‐free survival, month, mean ± SD	25.8 ± 16.3	19.2 ± 17.3	<0.001

Abbreviations: HBsAg, hepatitis B surface antigen; BCLC stage, Barcelona clinic liver cancer stage.

### 3.2 The PIV‐AFP status was correlated to elevated risk of prognosis

**FIGURE 2:**
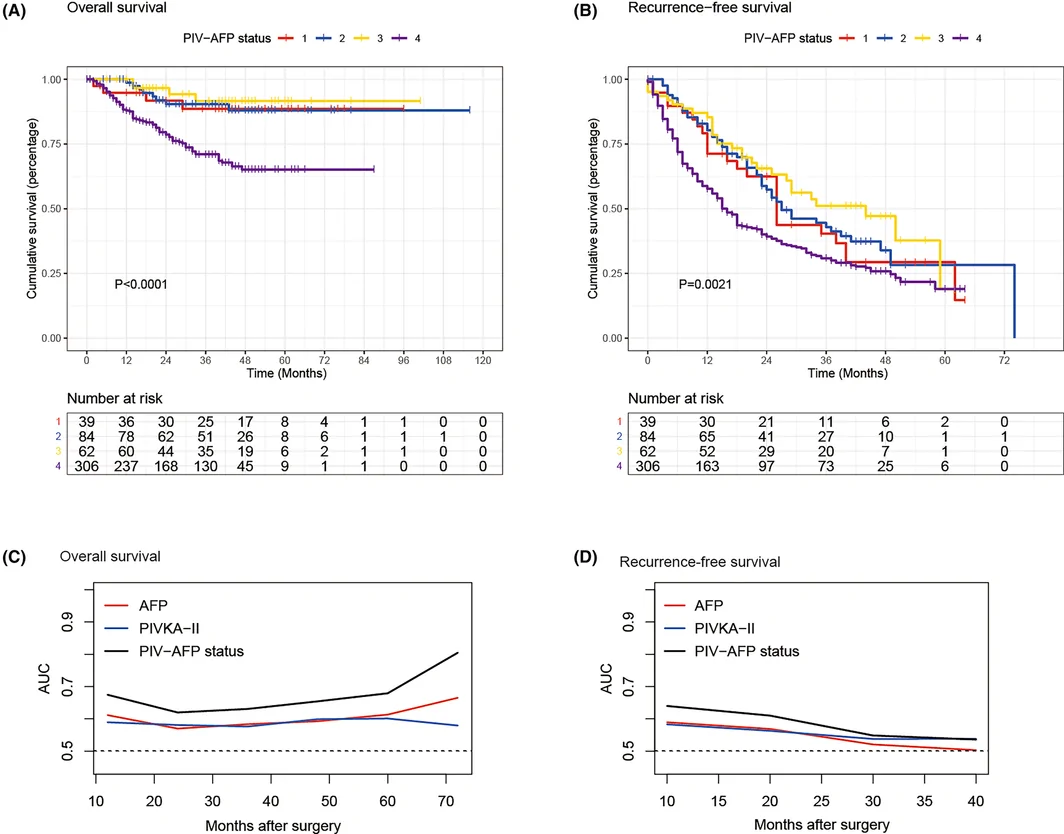
The predictive ability of PIV‐AFP status for OS and RFS in surgically treated HCC patients. (A, B) Kaplan–Meier curves of the patients after LH and OH for OS and RFS, PIV‐AFP status 4 had worse RFS and OS than the other 3 statuses. (C, D) The time‐dependent AUC curves of PIVKA‐II, AFP and PIV‐AFP status, the AUROC of the PIV‐AFP status was higher than that of the PIVKA‐II and the AFP for RFS and OS prediction.

Based on the preoperative serum PIVKA‐II and AFP secretion, the PIV‐AFP status was proposed, the patients were stratified into four statuses: PIVKA‐II (−) AFP (−) (status 1); PIVKA‐II (+) AFP (−) (status 2); PIVKA‐II (−) AFP (+) (status 3) and PIVKA‐II (+) AFP (+) (status 4). There were 320 patients in status 4 and 198 patients in status 1, 2, and 3. Compared with PIV‐AFP status 1, 2, 3, status 4 showed the following divergences: younger ages (*p* = 0.005); larger tumor size in diameters(*p* = 0.015); higher prevalence of poor tumor differentiation (*p* < 0.001), multiple tumor nodules (*p* = 0.016); higher prevalence of liver capsule invasion (*p* < 0.001) and MVI (*p* < 0.001); more advanced BCLC stage (*p* < 0.001), as well as higher rate of prophylactic targeted therapy postoperatively; the correlation between baseline characteristics with the PIV‐AFP status was demonstrated in Table 1. Among the 491 patients underwent liver resections (LH or OH), the patients with PIV‐AFP status 4 had worse 1‐, 3‐, 5‐year OS and RFS rates than those with the PIV‐AFP status 1, 2, and 3 (OS: 87.9%, 71.1%, 65.1% vs. 98.3%, 90.5%, 89.5%; RFS: 57.7%, 30.8%, 19% vs. 80.1%, 44.8%, 26.1%) (Figure 2A, B). Additionally, the PIV‐AFP status was with superior clinical efficacy in predicting the OS and RFS of surgically treated HCC patients through the results of time‐dependent AUROC analysis (Figure 2C, D). Moreover, the subgroup analyses using univariate and multivariate cox regression model were conducted based on gender (male or female), age (≤60 or > 60 years), tumor diameters (≤5 or >5 cm), tumor numbers (solitary or multiple), the conditions of the HBV infection, microvascular invasion, lymph node metastasis, liver capsule and adjacent organ invasions, and tumor differentiation. Although statistical significances were not obtained in all subgroups (the female patients, the patients with multiple tumors, etc.), the trends of poorer OS and RFS in the PIVKA‐II (+) and AFP (+) populations were in accordance with the previous survival curves. In addition, the PIV‐AFP status 4 remained as an independent risk factor in the prediction of the OS and RFS through the univariate and the multivariate analyses, together with several postoperative histological parameters (lymph node invasion, liver capsule invasion, and MVI) (Table 2, Table 3).

**TABLE 2:** Univariate and multivariate analyses to determine independent predictors of overall survival.

Variables	Univariate analysis			Multivariate analysis		
	HR	95% CI	*p* value	HR	95% CI	*p* value
Age, >60/ ≤60 (years)	0.8032	0.4824–1.3373	0.3996			
Gender, Female/Male	1.0245	0.5919–1.7733	0.931			
HBV	1.0838	0.6163–1.9060	0.7799			
Tumor size, >5/≤5 (cm)	2.4457	1.6425–3.6416	<0.0001	1.2567	0.8159–1.9358	0.2998
Tumor differentiation, undifferentiation‐poor/moderate‐well	2.6747	1.7749–4.0306	<0.0001	1.4147	0.9108–2.1975	0.1226
Tumor number, multiple/single	1.2076	0.7077–2.0605	0.4889			
Lymph node metastasis	3.5331	1.1192–11.1537	0.0314	1.4572	0.4395–4.8316	0.5381
Liver capsule invasion	2.5508	1.6828–3.8664	<0.0001	1.7029	1.1071–2.6192	0.0154
Microvascular invasion	4.3969	2.9484–6.5572	<0.0001	1.8716	1.0931–3.2047	0.0224
BCLC stage, B‐C/0‐A	2.9425	1.9328–4.4797	<0.0001	1.8098	0.861–3.8040	0.1175
PIV‐AFP status, 4/1,2,3	3.7885	2.2482–6.3840	<0.0001	2.5	1.4586–4.2848	0.0009

Abbreviations: HBV, hepatitis B virus; BCLC stage, Barcelona clinic liver cancer stage.

**TABLE 3:** Univariate and multivariate analyses to determine independent predictors of recurrence‐free survival.

Variables	Univariate analysis			Multivariate analysis		
	HR	95% CI	*p* value	HR	95% CI	*p* value
Age, >60/ ≤60 (years)	0.8292	0.6305–1.0907	0.1805			
Gender, Female/Male	0.8559	0.6245–1.1729	0.333			
HBV	1.0129	0.7451–1.3768	0.9349			
Tumor size, >5/≤5 (cm)	1.0129	0.7451–1.3768	0.9349			
Tumor differentiation, Undifferentiation‐Poor/Moderate‐Well	1.7114	1.3747–2.1306	<0.0001	1.2994	1.0289–1.6411	0.0278
Tumor number, Multiple/single	1.4024	1.0729–1.8332	0.0133	1.1834	0.8961–1.5630	0.2353
Lymph node metastasis	5.6982	2.5178–12.8959	<0.0001	2.5702	1.0882–6.0709	0.0313
Liver capsule invasion	1.5713	1.2619–1.9564	<0.0001	1.2331	0.9822–1.5482	0.0711
Microvascular invasion	2.3099	1.8413–2.8977	<0.0001	1.5223	1.136–2.0398	0.0049
BCLC stage, B‐C/ 0‐A	2.2254	1.7813–2.7803	<0.0001	1.681	1.3245–2.1334	<0.0001
PIV‐AFP status, 4/1,2,3	1.6619	1.3173–2.0967	<0.0001	1.2323	0.9644–1.5747	0.0349

Abbreviations: HBV, hepatitis B virus; BCLC stage, Barcelona clinic liver cancer stage.

### 3.3 The correlations between the PIV‐AFP status and surgical approaches

**FIGURE 3:**
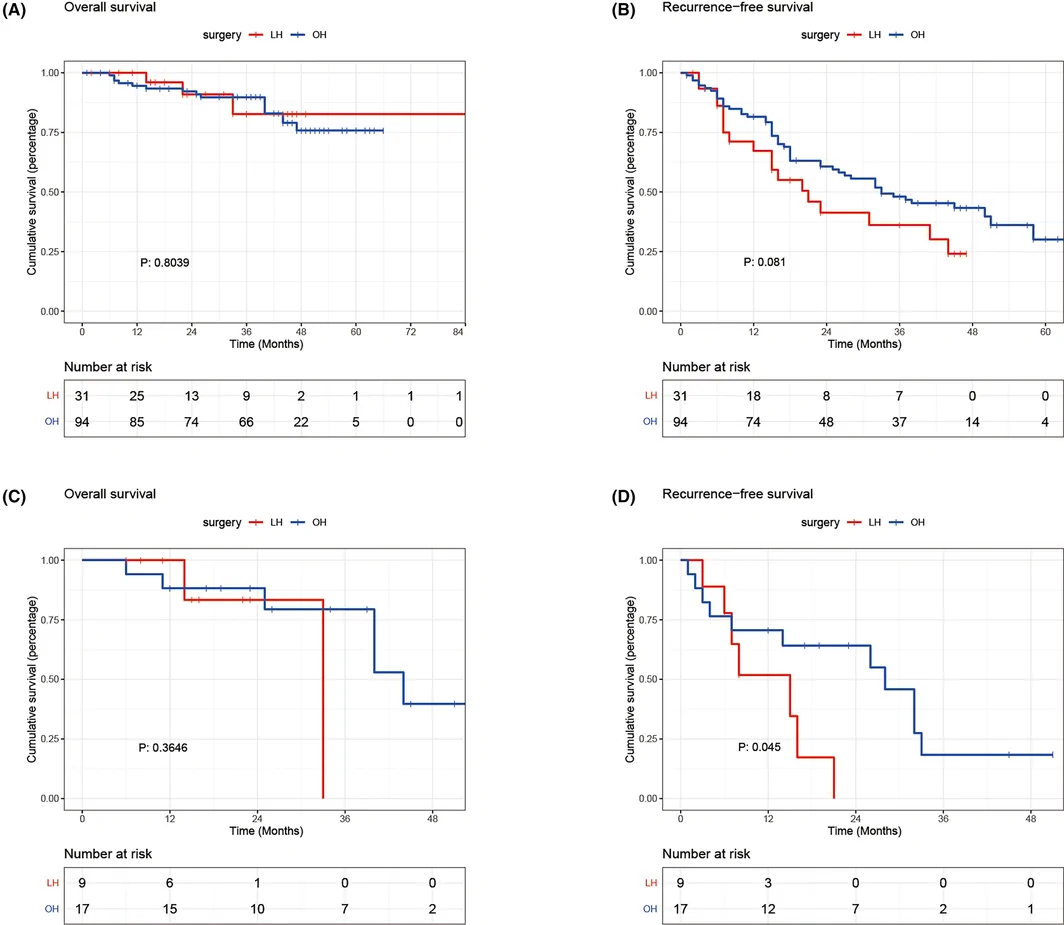
Differential survival of the patients with PIVKA‐II (+) and AFP (+) in BCLA 0‐A after LH and OH. (A) No difference between LH group and OH group for OS. (B) OH group had marginal significantly better RFS than LH group in this part of patients. Differential survival of the patients with PIVKA‐II (+), AFP (+), and MVI (+) in BCLA 0‐A after LH and OH. (C) No difference between LH group and OH group for OS. (D) Significantly decreased RFS was observed in LH group compared with OH group.

**FIGURE 4:**
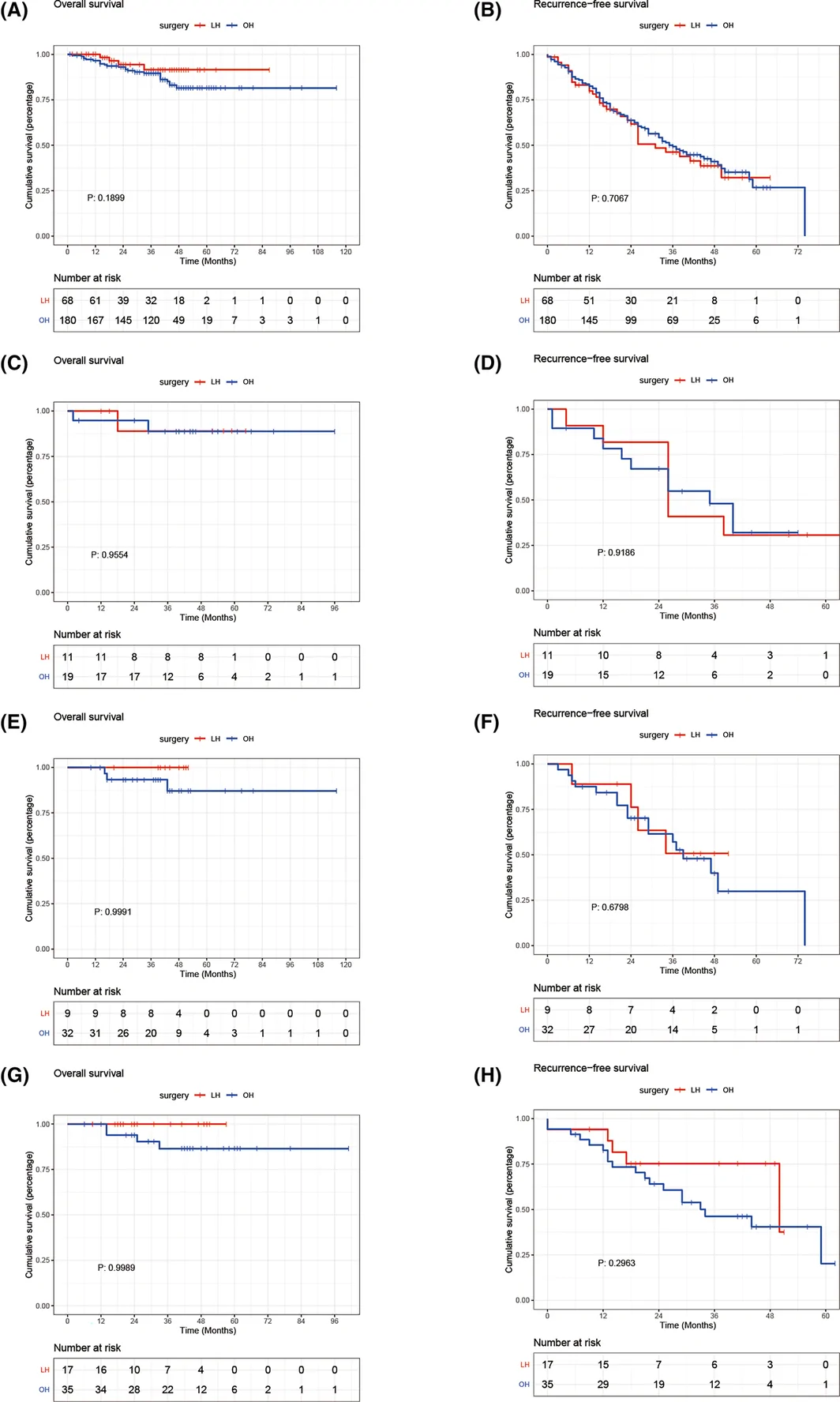
Patient's outcome in BCLC 0‐A for different surgical approaches (LH, laparoscopic hepatectomy; OH, open hepatectomy). (A, B) OS and RFS in all BCLC 0‐A HCC patients. (C, D) OS and RFS in BCLC 0‐A patients with PIV‐AFP status 1. (E, F) OS and RFS in BCLC 0‐A patients with PIV‐AFP status 2. (G, H) OS and RFS in BCLC 0‐A patients with PIV‐AFP status 3.

All the 248 patients staged in the BCLC 0‐A were stratified into laparoscopic hepatectomy (LH) group and open hepatectomy (OH) group; no difference was observed in the survival between LH and OH group (Figure 4A,B). The patients were subsequently subdivided according to the PIV‐AFP status; the OS and RFS of the patients after LH or OH in different PIV‐AFP statuses were measured. Interestingly, the patients with PIVKA‐II (+) and AFP (+) presented marginal significantly decreased RFS after the LH compared with the OH (median RFS: LH:15 months vs. OH: 25.5 months, *p* = 0.081) (Figure 3B); while there were no significant differences in the OS (Figure 3A) and in the other PIV‐AFP statuses (Figure 4C–H). To further reduce the potential selection bias of this retrospective study, a propensity score‐matched analysis was conducted, 23 patients in LH group and 57 patients in OH group were included (Table 4), after propensity score matching, the BCLC 0‐A HCC patients with PIV‐AFP status 4 underwent LH still presented marginal significantly declined RFS compared with those underwent OH (Figure S1). According to the previous study *14*, the preoperative serum PIVKA‐II value was related to the MVI. In this study, the differences of the RFS between the LH and OH groups could be affected by the presence of MVI; therefore, the patients with PIVKA‐II (+) and AFP (+) were further separated into the MVI (−) and MVI (+) subgroups; similarly, the BCLC stage A patients with positive MVI in the status 4 were with significantly worse RFS after LH than OH (*p* = 0.045) (Figure 3D), no significant decrease in the OS was observed (Figure 3C). To further assess the effects of surgical approaches on the RFS of BCLA 0‐A HCC patients with PIV‐APF status 4, we conducted multivariate analysis using cox regression model, the results suggested that LH remained a marginal risk factor for the RFS (HR: 1.5846, *p* = 0.0564) while MVI positivity showed the strongest predictive ability to inferior RFS (Table S1).

**TABLE 4:** Correlation of characteristics with surgical approaches (LH or OH) for the 80 BCLC 0‐A patients with PIV‐AFP status 4 after PSM.

Characteristics	Surgical approach		*p* value
	LH (*n* = 23)	OH (*n* = 57)	
Age, year, *n* (%)			
≤ 60	19 (82.6%)	44 (77.2%)	0.592
> 60	4 (17.4%)	13 (22.8%)	
Gender, *n* (%)			
Male	18 (78.3%)	45 (78.9%)	0.946
Female	5 (21.7%)	12 (21.1%)	
HBV (positive), *n* (%)	21 (91.3%)	51 (89.5%)	0.805
Poor tumor differentiation, *n* (%)	12 (52.2%)	24 (42.1%)	0.413
Multiple tumors, *n* (%)	4 (17.4%)	6 (10.5%)	0.401
Liver capsule invasion, *n* (%)	10 (43.5%)	26 (45.6%)	0.862
Microvascular invasion, *n* (%)	8 (34.8%)	11 (19.3%)	0.141
Overall survival, month, mean ± SD	28.2 ± 19.2	36.2 ± 16.5	0.062
Recurrence‐free survival, month, mean ± SD	21.0 ± 15.9	29.3 ± 18.0	0.057

Abbreviations: HBV, hepatitis B virus; LH, laparoscopic hepatectomy; OH, open hepatectomy.

## 4 DISCUSSION

The present study indicated that different preoperative PIVKA‐II and AFP secretion modes were related to the prognosis of the surgically treated HCC patients; 518 out of 618 HCC patients were enrolled in this study, among which 388 patients were received open anatomical hepatectomy and 103 underwent laparoscopic anatomical hepatectomy (Figure 1). Those with elevated levels of both the PIVKA‐II and AFP had higher prevalence of recurrences and worse outcomes. Meanwhile, the early HCC (BCLC 0‐A) patients with PIVKA‐II (+) and AFP (+) were associated with increased risks of postoperative recurrences, especially those with positive MVIs. Therefore, an assessment using both biomarkers may be helpful in predicting the prognosis of HCC and selecting appropriate surgical approaches for liver resections.

Since the association between the elevated serum PIVKA‐II levels and the advancing tumor characteristics was first reported in 1994 by Japanese researchers.^26^ A growing number of studies have validated that the higher level of PIVKA‐II in serum or in tissue is related to several crucial factors which affect the outcome of the HCC patient (larger tumor size, more frequent intrahepatic metastasis, major vessel invasion, and earlier recurrence after treatment).^26^, ^27^, ^28^, ^29^, ^30^ Despite the widespread application of the PIVKA‐II as a diagnostic tool and a prognostic predictor in the Asian population, it is not well‐established in the Western world. In comparison with the PIVKA‐II, the clinical efficacy of the AFP is under constant suspicion; one Korean study in 2009 even presented that the serum PIVKA‐II level was an independent risk factor to the outcome of HBV related HCC while the AFP was not.^31^ In the majority scenario, the combination of these two tumor markers is verified to be beneficial in predicting the survival of HCC both in the Asian^32^ and Caucasian^33^ populations. Moreover, in locally advanced HCC, the treatment responses of the PIVKA‐II and AFP show a predictive ability for progression‐free survival (PFS) and OS.^34^ In the present study, we divided the HCC patients according to the serum PIVKA‐II and AFP levels into four statuses: P (−) A (−), P (+) A (−), P (−) A (+), and P (+) A (+). The patients with P (+) A (+) were related to significantly increased rates of multiple tumors, liver capsule and microvascular invasions, as well as greater tumor sizes; the OS and RFS were likewise worse compared to the other statuses, in accordance with the earlier studies.^33^, ^34^

There is accumulating evidence indicating that the PIVKA‐II is correlated to the malignant biological behavior of HCC. The previous in vitro study suggested that PIVKA‐II acts as an autologous mitogen in three different HCC cell lines, which induces cell proliferation through the Janus kinase 1‐ Signal transducer and activator of transcription 3 (JAK1‐STAT3) signaling pathway.^35^ The subsequent study demonstrated that PIVKA‐II induces both proliferation and migration of the human umbilical vein endothelial cells (HUVEC) by activating the Kinase insert domain receptor‐Phospholipase C‐γ‐Mitogen‐activated protein kinase (KDR‐PLC‐γ‐MAPK) pathway, suggested that PIVKA‐II is a novel type of vascular endothelial growth factor (VEGF) which promotes angiogenesis of HCC.^36^ More recently, a study by Suzuki et al^37^ showed that hypoxia leads to the cessation of DCP secretion through hypophosphorylation of mammalian target of rapamycin (mTOR), which activates the epithelial–mesenchymal transition (EMT) and promotes HCC metastasis. These mechanisms of PIVKA‐II reinforce our results that more frequent recurrences and worse outcomes were observed in the patients with positive PIVKA‐II and AFP.

Liver resection remains the principal treatment for liver cancer, especially the HCC at an early stage; as the first LH was reported by Reich et al,^38^ the benefits of the laparoscopic approach in the treatment of HCC have been recognized by surgeons and is increasingly taking the place of the conventional OH. The kernel of attaining optimal long‐term survival after hepatectomy is the appropriate oncological resection plane and the negative margin; however, it is more challenging to get a proper resection plane in LH compared with OH due to the loss of touch.^39^ Thus far, little conclusion is drawn based on the randomized controlled trial (RCT) comparing the survival between LH and OH. Considering the controversy on LH, to improve the surgical outcome, the patient selection for laparoscopic approach in liver resection is essential. The presence of major vascular structure invasion is a contraindication of LH^23^; apart from this, the MVI was reported as an independent risk factor to RFS.^24^ In this study, the patients with elevated PIVKA‐II and AFP had worse RFS in the LH group than in the OH group; on account of this result, the LH should be applied with caution in this part of HCC patients.

Interestingly, the strong correlation between PIV‐AFP status and MVI was observed, the PIV‐AFP status 4 was an independent risk factor for MVI (HR, 2.4; 95% CI, 1.6–3.8; *p* < 0.0001), in line with the previous studies presenting the predictive value of the high PIVKA‐II level.^12^, ^14^, ^40^ Moreover, the areas under ROC of preoperative PIVKA‐II and PIV‐AFP status to predict MVI were comparable to several clinical and histological parameters (Tumor size, differentiation, and liver capsule invasion).

This study is noticeable for several reasons. Firstly, this was so far the largest single‐center cohort in assessing the prognostic value of PIVKA‐II to HCC patients, including 518 histologically diagnosed participants, another large multicenter cohort (1194 participants) reported by Yang et al mainly focused on the diagnosis of HCC in patients with chronic hepatitis B (CHB)^41^; the proposed PIV‐AFP status also showed optimal effectiveness in predicting long‐term outcome. Secondly, to the best of our knowledge, this was the first study evaluating the relationship between the serum PIVKA‐II level and the surgical approach for liver resection in HCC patients; the results might be helpful in the patient selection for laparoscopic hepatectomy.

There are several limitations despite the promising results: (1) indeed, this was a retrospective study, the unknown variables may confound the result; thus, a prospective study including multiple medical centers needs to be conducted; (2) the population in subgroups were small to carry out a solid conclusion; therefore, larger sample size and external validation are required in the subsequent study; (3) only serum levels of the tumor markers were assessed; as suggested in the previous studies,^14^, ^40^ the tissue PIVKA‐II expression measured by immunohistochemical (IHC) staining was more accurate than the serum level; in the future study, the immunostaining of the HCC sample needs to be applied.

## 5 CONCLUSION

A preoperative PIV‐AFP status was a validated prognostic marker for HCC after surgical treatment. PIV‐AFP status was a surrogate selection criterion for liver resection in HCC patients, LH should be conducted with caution in the PIVKA‐II (+) AFP (+) population. PIV‐AFP status was an independent risk factor for histological MVI.

## AUTHOR CONTRIBUTIONS

**Yunshi Cai:** Conceptualization (equal); data curation (equal); formal analysis (equal); writing – original draft (equal). **Kunlin Xie:** Conceptualization (equal). **Mohammad Natheir Adeeb Alhmoud:** Software (supporting). **Tian Lan:** Conceptualization (equal); data curation (equal); methodology (equal). **Haifeng Wan:** Data curation (equal). **Die Hu:** Data curation (equal). **Ling Lan:** Data curation (equal). **Chang Liu:** Conceptualization (equal); funding acquisition (equal); investigation (equal); writing – review and editing (equal). **Hong Wu:** Conceptualization (equal); funding acquisition (equal).

## Supporting information


Figure S1

Table S1

## Data Availability

The data that support the findings of this study are available from the corresponding author upon reasonable request.
