# Supplementary figures and images for: Effect of PIVKA‐II and AFP secretion status on early recurrence of hepatocellular carcinoma after open and laparoscopic surgery
Source: Cancer Med. 2023 Aug 18;12(17):17866–77. doi: 10.1002/cam4.6422 (PMC10523999; doi:10.1002/cam4.6422)

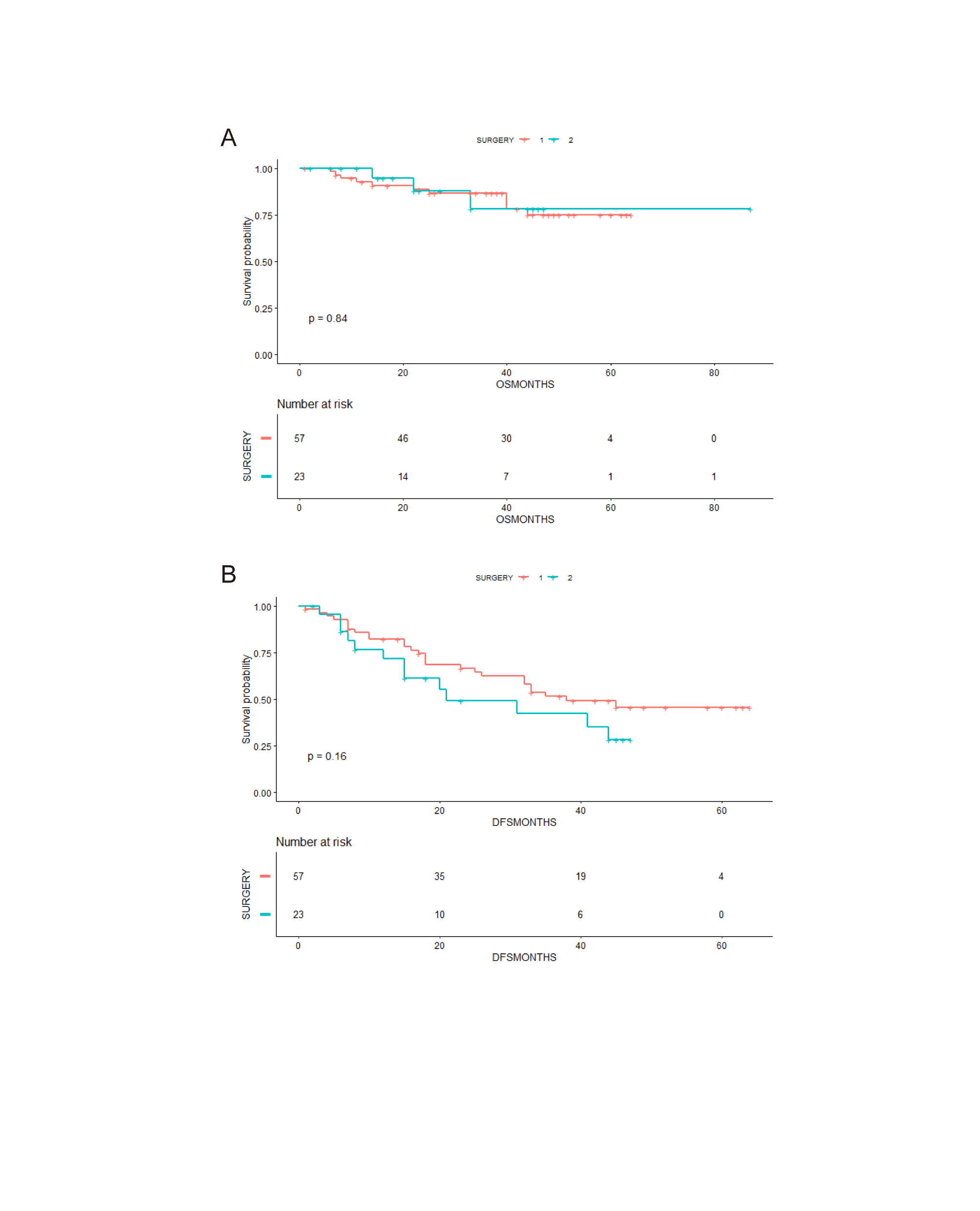

Supplement: Supplementary file 1 — Figure S1 [file CAM4-12-17866-s002.tif]
