# Supplementary material for: Effect of PIVKA‐II and AFP secretion status on early recurrence of hepatocellular carcinoma after open and laparoscopic surgery
Source: Cancer Med. 2023 Aug 18;12(17):17866–77. doi: 10.1002/cam4.6422 (PMC10523999; doi:10.1002/cam4.6422)
Supplement: Supplementary file 2 — Table S1 [file CAM4-12-17866-s001.docx]

**Supplementary table 1. Risk factors for recurrence-free survival in BCLC 0-A HCC patients with PIV-AFP status 4**

| **Variables** | **Multivariate analysis** | | |
| --- | --- | --- | --- |
|  | **HR** | **95%CI** | **P value** |
| Age, >60/ ≤60 (years) | 1.1233 | 0.3705-1.4172 | 0.3466 |
| Gender, Female/Male | 0.8249 | 0.4045-1.6823 | 0.5965 |
| HBV | 0.7662 | 0.4053-1.4484 | 0.4123 |
| Tumor size, >5/≤5 (cm) | 1.0776 | 0.1353-8.5779 | 0.9437 |
| Tumor number, Multiple/ single | 0.9342 | 0.4787-1.8232 | 0.8418 |
| Tumor differentiation, Undifferentiation-Poor/Moderate-Well | 1.3357 | 0.8244-2.1644 | 0.2398 |
| Liver capsule invasion | 0.68 | 0.4057-1.1397 | 0.1433 |
| Microvascular invasion | 2.3827 | 1.3111-4.3303 | 0.0044 |
| Surgery, LH/OH | 1.5846 | 0.9987-1.9652 | 0.0564 |

Abbreviations: HBV, hepatitis B virus; LH, laparoscopic hepatectomy; OH, open hepatectomy
